# Spin Glasses in a Field Show a Phase Transition Varying the Distance among Real Replicas (And How to Exploit It to Find the Critical Line in a Field)

**DOI:** 10.3390/e22020250

**Published:** 2020-02-22

**Authors:** Maddalena Dilucca, Luca Leuzzi, Giorgio Parisi, Federico Ricci-Tersenghi, Juan J. Ruiz-Lorenzo

**Affiliations:** 1 Dipartimento di Fisica, Sapienza Universitá di Roma, P.le A. Moro 2, I-00185 Roma, Italy; maddyemario@hotmail.it; 2 CNR, Nanotec, Rome unit, P.le A. Moro 2, I-00185 Roma, Italy; 3 INFN, Sezione di Roma I, P.le A. Moro 2, I-00185 Roma, Italy; 4 Departamendo de Física and ICCAEx, Universidad de Extremadura, 06006 Badajoz, Spain; 5 Instituto de Biocomputación y Física de los Sistemas Complejos (BIFI), 50018 Zaragoza, Spain

**Keywords:** disordered systems, spin glasses, mean field, phase transitions, numerical simulations

## Abstract

We discuss a phase transition in spin glass models that have been rarely considered in the past, namely, the phase transition that may take place when two real replicas are forced to be at a larger distance (i.e., at a smaller overlap) than the typical one. In the first part of the work, by solving analytically the Sherrington-Kirkpatrick model in a field close to its critical point, we show that, even in a paramagnetic phase, the forcing of two real replicas to an overlap small enough leads the model to a phase transition where the symmetry between replicas is spontaneously broken. More importantly, this phase transition is related to the de Almeida-Thouless (dAT) critical line. In the second part of the work, we exploit the phase transition in the overlap between two real replicas to identify the critical line in a field in finite dimensional spin glasses. This is a notoriously difficult computational problem, because of considerable finite size corrections. We introduce a new method of analysis of Monte Carlo data for disordered systems, where the overlap between two real replicas is used as a conditioning variate. We apply this analysis to equilibrium measurements collected in the paramagnetic phase in a field, h>0 and $T_{c}\left(h\right)<T<T_{c}\left(h=0\right)$, of the d=1 spin glass model with long range interactions decaying fast enough to be outside the regime of validity of the mean field theory. We thus provide very reliable estimates for the thermodynamic critical temperature in a field.

## 1. Introduction

The study of spin glass models in an external field started more than 40 years ago [1], but has demonstrated to be an extremely challenging problem. The results of the many numerical simulations performed over the last three decades [2,3,4,5,6,7,8,9,10,11,12,13,14,15,16,17,18,19,20,21] have been inconclusive and often interpreted in contradicting ways. The main reason for this difficulty seems to rely on the large finite size corrections that spin glasses have in the presence of an external magnetic field, which make it very difficult to extract the thermodynamic behavior.

The presence of strong finite size corrections has been known since the very first numerical simulations [2,3,4,5]. However, only recently has it been possible to obtain a quantitative measure of it by running Monte Carlo simulations in a model whose critical behavior is known analytically [22]. Indeed, by using the cavity method [23], spin glass models on random graphs can be analytically solved in the paramagnetic phase, and the critical dAT line separating the paramagnetic and the spin glass phases can be computed exactly. However, a standard analysis of Monte Carlo measurements taken from a spin glass model in a field defined on a random graph fail to correctly identify the critical temperature [22]. A possible explanation of this surprising finding comes from the observation made in [24]: that mean values of the observables used in the Monte Carlo analysis are dominated by a minority of atypical measurements (e.g., atypical in the value of the mean overlap *q*). A better analysis focusing on typical measurements can correctly identify the critical point predicted analytically.

A similar problem in the analysis of Monte Carlo data measured from a spin glass model in a field was found in [16], where it was found that a standard finite size scaling analysis was unable to identify the correct critical point in the presence of a field, while the same analysis works perfectly in the absence of the external field. The main source of fluctuations, leading to the dominance of atypical measurements, was identified in the value of the 4-points correlation at a large distance, i.e., $q^{2}$, where $q=\sum_{i}s_{i}t_{i}/N$ is the overlap between the two simulated real replicas s and t. This value enters in the Fourier transform of the correlation function at zero wavelength (k=0). By performing a new analysis that avoids the use of the k=0 component, and restricts to k∈{2π/L,4π/L}, it was possible to clearly identify a phase transition in a field [16].

Subsequent works [17,18,21] confirmed with more statistics and in a broader class of spin glass models in a field that the main source of these considerable finite size corrections is in the behavior of atypical samples and/or atypical measurements. In summary, what seems to happen in spin glass models under the effect of an external field is the following. Even for the largest systems that can be simulated with present computer facilities, the probability distribution of the overlap is very broad and shows an exponential tail extending in the region of small and even negative overlaps. This tail is clearly a finite size effect, that must disappear in the thermodynamic limit. However, the measurements corresponding to these atypically small values of the overlap tend to dominate the average in samples of finite size and to hide the behavior of the vast majority of measurements.

In order to extract the typical behavior, in [21], all the measurements have been divided in 10 deciles according to a conditioning variate, i.e., essentially the overlap among the simulated replicas. The results clearly show that the behavior of the first (or the last) decile is definitely different from the median behavior. Moreover, the behavior of the median decile is the one showing the least finite size effects; therefore, it is likely to approach the thermodynamic behavior faster in *N*. From measurements based on data relative to this median decile in *q*, one can observe a clear phase transition that was impossible to identify taking the unconditional average on all the measurements.

Thus, the general picture that emerges from several different analyses carried out until now is that the behavior of spin glasses in a field may strongly depend on the specific value of the overlap between the real replicas simulated. This seems to be true even in the paramagnetic phase, $T>T_{c}\left(h\right)$, where asymptotically, in the large *N* limit, the overlap takes a unique value, $q=q_{\mathrm{EA}}$, with high probability.

Therefore, it is seems natural to us to consider a spin glass model in a field with two real replicas constrained to take an overlap equal to *q*, and check how much the behavior of the model depends on the value of *q*. Moreover, fixing the overlap between the two real replicas to *q*, one has the advantage that fluctuations in *q* are suppressed, and this might only produce a better signal-to-noise ratio in the analysis of numerical data.

In the following, we provide detailed information on the above points. In Section 2, we solve a mean field model for spin glasses in a field and show indeed that a phase transition takes place if the overlap between the real replicas is made small enough, and this can be connected to the critical dAT line. In Section 3, we analyze numerical data for a finite-dimensional spin glass in a field conditioning on the overlap between the two simulated replicas, finding, indeed, a phase transition towards a spin glass phase. This conditioned analysis provides reliable estimates for the location of the critical dAT line $T_{c}\left(h\right)$.

## 2. Phase Transition Varying the Overlap between Two Real Replicas in a Solvable Mean Field Model

Our interest is in understanding what happens to the paramagnetic phase of a disordered model when two real replicas are coupled and forced to be at an atypical overlap value. In order to be consistent with previous literature [25], in this Section, we call $p_{d}$ the overlap at which the two real replicas are forced to be. In particular, we would like to uncover possible phase transitions upon changing $p_{d}$ and connect these with the phase transition on the dAT line.

In order to work with a solvable mean field model, we consider the so-called truncated model that describes correctly the Sherrington-Kirkpatrick (SK) model close to its critical point $\left(T=T_{c}=1,h=0\right)$. The equations of two replicas constrained to be at overlap $p_{d}$ were already considered in [26]. However, in that work, the authors focused mainly on the Replica Symmetry Breaking (RSB) solutions, while we are mostly interested in identifying eventual phase transitions taking place in the paramagnetic phase when $p_{d}<q_{\mathrm{EA}}$.

### 2.1. The Truncated Model

The truncated model is an expansion of the free energy of the SK model in powers of the *Q* matrix up to the fourth order term, which is responsible for the breaking of the replica symmetry [27]. Under this “truncated” approximation, the free energy reads

(1) $$F=\tau \langle q^{2}\rangle -\frac{1}{3}\left[2\langle q\rangle \langle q^{2}\rangle +\int_{0}^{1}dx\;q\left(x\right)\int_{0}^{x}dz\;{\left[q\left(x\right)-q\left(z\right)\right]}^{2}\right]+\frac{1}{4}y\langle q^{4}\rangle +h^{2}\langle q\rangle \;,$$

where $\langle q^{k}\rangle \equiv \int_{0}^{1}q{\left(x\right)}^{k}dx$, being q(x) the Parisi order parameter, $\tau =1-T/T_{c}\left(h=0\right)=1-T$, and y=2/3 for the SK model. The function q(x) extremizing the truncated free energy has been computed in [27] and reads

(2) $$\begin{array}{ccc} q(x)= & q_{\mathrm{EA}}\left(\tau ,h\right) & \mathrm{for}\;\tau \leq \tau_{c}\left(h\right)\;, \end{array}$$

(3) $$\begin{array}{ccc} q(x)= & \left\{\begin{array}{cc} q_{\min}\left(h,y\right) & 0\leq x\leq 3yq_{\min}\;,\\ \frac{x}{3y} & 3yq_{\min}<x<3yq_{\max}\;,\\ q_{\max}\left(\tau ,y\right) & 3yq_{\max}\leq x\leq 1\;, \end{array}\right. & \mathrm{for}\;\tau >\tau_{c}\left(h\right)\;, \end{array}$$

where

(4) $$q_{\min}\left(h,y\right)={\left(\frac{h^{2}}{2y}\right)}^{1/3}\;,\;q_{\max}\left(\tau ,y\right)=\frac{1-\sqrt{1-6y\tau }}{3y}\;,$$

and $q_{\mathrm{EA}}\left(\tau ,h,y\right)$ satisfies

(5) $$h^{2}+2\tau q_{\mathrm{EA}}-2q_{\mathrm{EA}}^{2}+yq_{\mathrm{EA}}^{3}=0\;.$$

The dAT line signaling the onset of the RSB phase can be obtained by imposing the condition $q_{\min}\left(h,y\right)=q_{\max}\left(\tau ,y\right)$ and, to leading order, is given by $\tau_{c}\left(h,y\right)={\left(h^{2}/\left(2y\right)\right)}^{1/3}$ or $h_{c}\left(\tau ,y\right)=\sqrt{2y}\;\tau^{3/2}$. It is worth noticing that, in the paramagnetic phase, i.e., for $\tau <\tau_{c}\left(h,y\right)$ or $h>h_{c}\left(\tau ,y\right)$, the order of the three overlaps just defined is $q_{\max}\left(\tau ,y\right)<q_{\mathrm{EA}}\left(\tau ,h,y\right)<q_{\min}\left(h,y\right)$.

### 2.2. The Model with Constrained Replicas

The case of two real replicas constrained to have overlap $p_{d}$ has been considered in [26]. A more complex order parameter that involves two matrices *Q* and *P* is required, where *Q* (resp. *P*) describes the overlaps between copies of the same (resp. different) real replica(s). Matrices *Q* and *P* are parametrized as usual via the Parisi functions q(x) and p(x), thus obtaining the following free energy

(6) $$\begin{array}{cc} F= & \tau \left[\langle q^{2}\rangle +\langle p^{2}\rangle -p_{d}^{2}\right]-\frac{1}{3}[2\langle q\rangle \langle q^{2}\rangle +\int_{0}^{1}dx\;q\left(x\right)\int_{0}^{x}dz\;{\left[q\left(x\right)-q\left(z\right)\right]}^{2}+6\langle pq\rangle \left(\langle p\rangle -p_{d}\right)\\ \; & +3\int_{0}^{1}dx\;q\left(x\right)\int_{0}^{x}dz\;{\left[p\left(x\right)-p\left(z\right)\right]}^{2}]+\frac{y}{4}\left[\langle q^{4}\rangle +\langle p^{4}\rangle -p_{d}^{4}\right]+h^{2}\left[\langle q\rangle +\langle p\rangle -p_{d}\right]\;. \end{array}$$

We consider $p_{d}$ as a free parameter that we want to change in order to test for eventual phase transitions when $p_{d}$ becomes small. Thus, the free energy needs to be extremized only with respect to q(x) and p(x). The corresponding equations are the following

(7) $$\begin{array}{cc} \frac{\delta F}{\delta q(x)} & =2\left(\tau -\langle q\rangle \right)q\left(x\right)+2\left(p_{d}-\langle p\rangle \right)p\left(x\right)-\int_{0}^{x}dz\;{\left[q\left(x\right)-q\left(z\right)\right]}^{2}-\int_{0}^{x}dz\;{\left[p\left(x\right)-p\left(z\right)\right]}^{2}+yq^{3}\left(x\right)+h^{2}=0\;,\\ \frac{\delta F}{\delta p(x)} & =2\left(\tau -\langle q\rangle \right)p\left(x\right)+2\left(p_{d}-\langle p\rangle \right)q\left(x\right)-2\int_{0}^{x}dz\;\left[q\left(x\right)-q\left(z\right)\right]\left[p\left(x\right)-p\left(z\right)\right]+yp^{3}\left(x\right)+h^{2}=0\;. \end{array}$$

Taking some derivatives and doing a little bit of algebra, one can prove in general the following statements [26]:

- If $q^{\prime }\left(x\right)=0$, then $p^{\prime }\left(x\right)=0$.
- If $q^{\prime }\left(x\right)\neq 0$ and $p^{\prime }\left(x\right)=0$, then $q\left(x\right)=\frac{x}{3y}$.
- If p(z)=q(z)∀z≤x, then either $q^{\prime }\left(x\right)=0$ or $q\left(x\right)=\frac{2x}{3y}$.

We will investigate different quite general ansatz for q(x) and p(x) compatible with these conditions, but we do not find useful to write down the most general ansatz compatible with the constraints because it is rather cumbersome. We prefer to add complexity to the solution step by step.

### 2.3. Replica Symmetry (RS) Solutions

We start with the RS solution q(x)=q and p(x)=p, which certainly holds above the dAT line, when the constraining overlap $p_{d}$ is close to the typical value for the overlap $q_{\mathrm{EA}}\left(\tau ,h,y\right)$. The equations to be solved are

(8) $$\begin{array}{ccc} h^{2}+2p_{d}q+2\tau p-4qp+yp^{3} & = & 0\;, \end{array}$$

(9) $$\begin{array}{ccc} h^{2}+2\tau q+2p_{d}p-2q^{2}-2p^{2}+yq^{3} & = & 0\;. \end{array}$$

These equations admit a symmetric solution $p=q=q_{\mathrm{EA}}\left(p_{d},\tau ,h,y\right)$ with the latter defined by

(10) $$h^{2}+2\left(\tau +p_{d}\right)q_{\mathrm{EA}}-4q_{\mathrm{EA}}^{2}+yq_{\mathrm{EA}}^{3}=0\;.$$

The $q_{\mathrm{EA}}$ overlap in the model with two constrained replicas is related to the one in the model with a single replica through the following transformation of parameters: $\tau \to \frac{\tau +p_{d}}{2},h\to \frac{h}{\sqrt{2}},y\to \frac{y}{2}$. In other words,

(11) $$q_{\mathrm{EA}}\left(p_{d},\tau ,h,y\right)=q_{\mathrm{EA}}\left(\frac{\tau +p_{d}}{2},\frac{h}{\sqrt{2}},\frac{y}{2}\right)\;.$$

From this observation, it is easy to obtain the condition under which the RS symmetric solution $q=p=q_{\mathrm{EA}}\left(p_{d},\tau ,h,y\right)$ is stable with respect to a solution still symmetric, q(x)=p(x), but breaking the replica symmetry:

(12) $$q_{\max}\left(\frac{\tau +p_{d}}{2},\frac{y}{2}\right)<q_{\min}\left(\frac{h}{\sqrt{2}},\frac{y}{2}\right)\;.$$

This equation defines an upper bound on $p_{d}$, because $q_{\max}\left(\tau ,y\right)$ is monotonously increasing in τ. Hereafter, we will always work in the range of $p_{d}$ satisfying Equation (12). For example, for τ=0.1, h=0.1, and y=2/3, the bound reads $p_{d}<0.253171$ (these values for τ, *h*, and *y* define a point in the paramagnetic phase of the SK model and will be used as an example in the rest of this section). In the paramagnetic phase, fixing $p_{d}=q_{\mathrm{EA}}\left(\tau ,h,y\right)$, that is constraining the real replicas to the typical value, the bound in Equation (12) is always satisfied.

Lowering enough the value of $p_{d}$, the symmetry p(x)=q(x) can spontaneously break down. At the RS level, this actually corresponds to the free energy being extremized by a solution with p≠q. Such a phase transition takes place at $p_{d}=p_{d}^{*}$ where $p_{d}^{*}\left(\tau ,h,y\right)$ can be obtained from the linearization of Equations (8)–(9) around the solution $p=q=q_{\mathrm{EA}}\left(p_{d},\tau ,h,y\right)$ and solves the following equation:

(13) $$p_{d}^{*}=\tau +\frac{3y}{2}{\left(q_{\mathrm{EA}}\left(p_{d}^{*},\tau ,h,y\right)\right)}^{2}\;.$$

**Figure 1:**
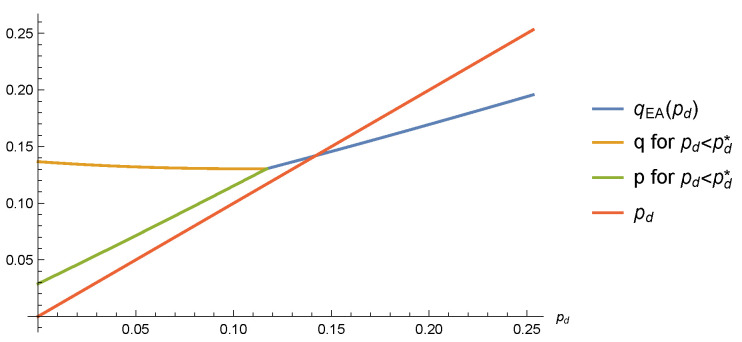
Parameters of the RS solutions versus $p_{d}$ in the case of τ=h=0.1 and y=2/3. The merging of the three curves takes place at $p_{d}=p_{d}^{*}\left(\tau ,h,y\right)=0.117033$, while the crossing between the two curves takes place at $q_{\mathrm{EA}}\left(\tau ,h,y\right)=0.141942$.

For $p_{d}\geq p_{d}^{*}$, we have $p=q=q_{\mathrm{EA}}\left(p_{d},\tau ,h,y\right)$, while for $p_{d}<p_{d}^{*}$ we have p<q (see Figure 1 for an example in the case τ=h=0.1 and y=2/3).

**Figure 2:**
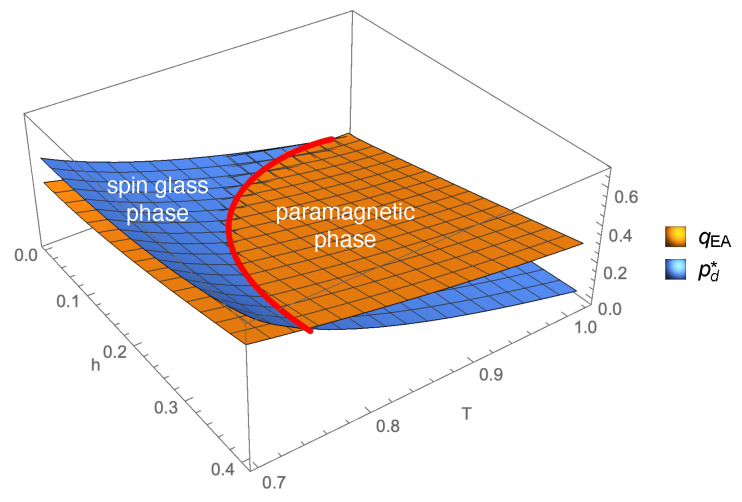
Values of $q_{\mathrm{EA}}\left(\tau ,h,y\right)$ and $p_{d}^{*}\left(\tau ,h,y\right)$ plotted in the (h,T=1-τ) plane for y=2/3. The red bold curve is the dAT line, separating the paramagnetic and the spin glass phases. $q_{\mathrm{EA}}$ and $p_{d}^{*}$ merge on the dAT line, while their values in the spin glass phase have no physical meaning. Below the blue surface in the paramagnetic phase, the p(x)=q(x) symmetry is broken.

The inequality $p_{d}^{*}\left(\tau ,h,y\right)<q_{\mathrm{EA}}\left(\tau ,h,y\right)$ that we can easily check in Figure 1 for τ=h=0.1 and y=2/3 is a general feature of the paramagnetic phase. In Figure 2, we show $q_{\mathrm{EA}}\left(\tau ,h,y\right)$ and $p_{d}^{*}\left(\tau ,h,y\right)$ for y=2/3, and we notice that the dAT line separating the paramagnetic and spin glass phases corresponds exactly to the locus where $q_{\mathrm{EA}}$ and $p_{d}^{*}$ coincide. Below the blue surface in the paramagnetic phase, the p(x)=q(x) symmetry is broken.

**Figure 3:**
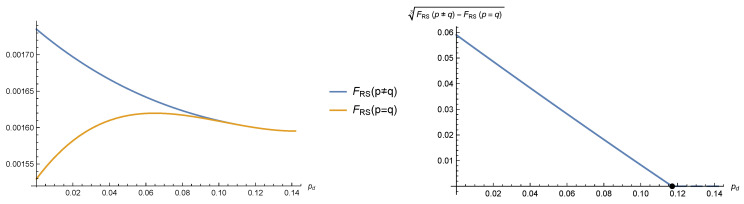
Free energies of the p=q and p≠q RS solutions for τ=h=0.1 and y=2/3. Below $p_{d}^{*}=0.117033$, the free energy of the p≠q solution is higher, and such a solution dominates over the symmetric one (**left panel**). The free energy difference goes as ${\left(p_{d}^{*}-p_{d}\right)}^{3}$, as can be seen in the (**right panel**), where the black dot marks the value of $p_{d}^{*}$.

In Figure 3, we show for τ=h=0.1 and y=2/3 the free energies of the p=q and p≠q solutions. Below $p_{d}^{*}=0.117033$, the free energy of the p≠q solution is higher, and such a solution dominates over the symmetric one (left panel). The free energy difference behaves as ${\left(p_{d}^{*}-p_{d}\right)}^{3}$, as can be seen from the right panel, where the black dot marks the value of $p_{d}^{*}$.

### 2.4. Replica Symmetry Breaking (RSB) Solutions in the Paramagnetic Phase

The next step is to show that, at $p_{d}^{*}$, where the p(x)=q(x) symmetry is spontaneously broken, and the replica symmetry spontaneously breaks. In order to show this, we have to search for solutions to the saddle point equations in Equation (7) with an RSB order parameter. We assume a single breaking point for both p(x) and q(x); i.e., we use the following ansatz:

(14) $$p\left(x\right)=\left\{\begin{array}{cc} p_{0} & 0\leq x<m\\ p_{1} & m<x\leq 1 \end{array}\right.\;q\left(x\right)=\left\{\begin{array}{cc} q_{0} & 0\leq x<m\\ q_{1} & m<x\leq 1 \end{array}\right.$$

We now have five saddle point equations to fix $p_{0},p_{1},q_{0},q_{1}$, and *m*. We looked numerically to their solutions, and we found that, beyond the RS solution $p_{0}=p_{1}=p$ and $q_{0}=q_{1}=q$, two other solutions exist:

1. a solution with $p_{0}≳p_{1}≃p$ and $q_{0}≲q_{1}≃q$, i.e., with the p(x) and q(x), respectively, very close to the RS corresponding overlaps *p* and *q*,
2. a solution with $p_{1}≃p<p_{0}=q_{0}<q_{1}≃q$, i.e., where $p_{1}$ and $q_{1}$ are close to the RS overlaps and at a small *x*, a mean overlap is roughly found $p_{0}=q_{0}≃\frac{p+q}{2}$.

We observe that, in both of these solutions, p(x) is a non-increasing function, since $p_{0}>p_{1}$. This may seem at odds with the standard interpretation of the hierarchical structure of states in the SK model, where q(x) is required to be a non-decreasing function. However, what is required for a correct physical interpretation of the states is the positivity of the matrix $\left(\begin{array}{cc} Q & P\\ P & Q \end{array}\right)$, which can be ensured if a decrease in the function p(x) is compensated by a larger increase in the function q(x), i.e., if $q_{1}-q_{0}>p_{0}-p_{1}$. We have checked that all the solutions found satisfy this criterion.

**Figure 4:**
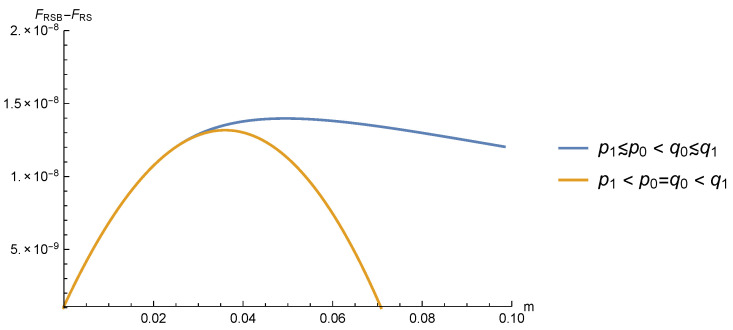
Difference between the one-step RSB (1RSB) free energies and $F_{\mathrm{RS}}$ for the two solutions with τ=h=0.1, y=2/3, and $p_{d}=0.05$. We notice that the difference is very small, but clearly non-zero. Moreover, the maximum is achieved for a rather small value of m, thus limiting the difference with respect to the RS solution to very small values of *x* (we remind the reader that, in both 1RSB solutions, $p_{1}≃p$ and $q_{1}≃q$).

We found that these one-step RSB (1RSB) solutions have a slightly better free energy with respect to the RS solution with p≠q. In Figure 4, we show for τ=h=0.1, y=2/3, and $p_{d}=0.05$ the difference between the 1RSB free energies and $F_{\mathrm{RS}}$ for the two solutions listed above. We notice that the difference is very small, but clearly non-zero. Moreover, the maximum is achieved for a rather small value of *m*, thus limiting the difference with respect to the RS solution to very small values of *x* (remember that, in both the 1RSB solutions, $p_{1}≃p$ and $q_{1}≃q$).

**Figure 5:**
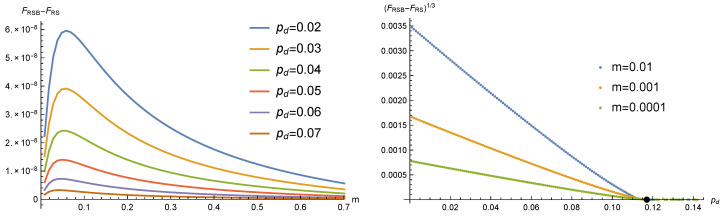
Difference between the dominating 1RSB free energy and $F_{\mathrm{RS}}$ as a function of *m* (**left**) and $p_{d}$ (**right**). The left panel shows that the location of the maximum of $F_{\mathrm{RSB}}$ slightly decreases when $p_{d}$ grows, but the main effect is that, for any *m* value, $F_{\mathrm{RSB}}$ tends to move toward $F_{\mathrm{RS}}$ when $p_{d}$ grows. The right panel shows that, for different *m* values, the free energy difference becomes zero very close to $p_{d}^{*}$, marked with a black dot. Note that data in the region close to $p_{d}^{*}$ may have some uncertainty due to the extremely small free energy differences, which are of the order $O(10^{-12})$.

Although the free energy difference between the two RSB solutions found is extremely small, the first one is to be preferred, where p(x)≃p and q(x)≃q (see Figure 4). We studied how the free energy difference between this solution and the RS one changes as the value of $p_{d}$ is varied. The results are shown in the left panel of Figure 5: the location of the maximum of $F_{\mathrm{RSB}}$ slightly decreases when $p_{d}$ grows, but the main effect is that, for any *m* value, $F_{\mathrm{RSB}}$ tends to move toward $F_{\mathrm{RS}}$ when $p_{d}\to p_{d}^{*}$ from below.

In order to better estimate the $p_{d}$ value where $F_{\mathrm{RSB}}$ and $F_{\mathrm{RS}}$ become equal, we plotted in the right panel of Figure 5 ${\left(F_{\mathrm{RSB}}-F_{\mathrm{RS}}\right)}^{1/3}$ as a function of $p_{d}$. This choice was dictated by the expectation that free energy differences are cubic as in the RS case above and in previous studies [26]. Since the location of the maximum in the left panel slightly changes with $p_{d}$, we show in the right panel three different values of *m*. We observe that all three curves extrapolate linearly to zero at a $p_{d}$ value very close to $p_{d}^{*}$, marked with a black dot. Note that data in the region close to $p_{d}^{*}$ may have some uncertainty due to the extremely small free energies difference, which are of the order $O(10^{-12})$.

We have verified that the same analysis also holds for other values of τ and *h*. We conclude that, in the paramagnetic phase of the SK model, when lowering the relative overlap between two real replicas, the system undergoes at $p_{d}^{*}$ a phase transition, where the main effect is the breaking of the p(x)=q(x) symmetry. Additionally, at the same critical point, or very close to it, the replica symmetry is broken.

The identification of the location $p_{d}^{*}$ of this phase transition in the paramagnetic phase is very useful in order to locate the dAT line given that the latter coincides with the condition $p_{d}^{*}=q_{\mathrm{EA}}$, which can be checked in numerical simulations. This is our aim in the next section.

## 3. Numerical Results in a Finite-Dimensional Spin Glass Model Varying the Overlap between Two Real Replicas

According to the analytical results derived in the previous section, a spin glass model in a paramagnetic phase with a non-zero field, i.e., with h>0 and $T_{c}\left(h\right)<T<T_{c}\left(h=0\right)$, is expected to undergo a phase transition to a spin glass phase as the overlap $p_{d}$ between two real replicas is decreased to a value $p_{d}^{*}$. Moreover, on the dAT line, the equality $p_{d}^{*}=q_{\mathrm{EA}}$ holds.

In this section, we analyze the numerical data collected in the paramagnetic phase of a spin glass model with a non-zero field with the aim of identifying $p_{d}^{*}$ and $q_{\mathrm{EA}}$. Given that we prefer to keep a different notation for the analytical critical point $p_{d}^{*}$ and the numerical estimate of the phase transition in *q*, we call the latter $q_{c}$ (but the reader should keep in mind that $q_{c}$ is the best numerical estimate for $p_{d}^{*}$).

### 3.1. Model and Numerical Simulations

Being interested in finite-dimensional spin glass models in a magnetic field, whose Hamiltonian is given by

(15) $$H\left(s\right)=-\sum_{i,j}J_{ij}s_{i}s_{j}-h\sum_{i}s_{i}\;,$$

we study a d=1 diluted spin glass model with long range interactions introduced in [15]. In this model, each spin interacts on average with six neighbors and the interactions are present with a probability depending on the distance, i.e., $J_{ij}=\pm 1$ with probability $P\left[J_{ij}\neq 0\right]\propto {\left|i-j\right|}^{-\rho }$. Changing the exponent ρ, the effective dimension of the model varies [15], and for $\rho >\rho_{U}=4/3$ the model is outside the range of validity of the mean field theory and thus presents a non-trivial critical behavior (as in a finite-dimensional spin glass model). The advantage of this model, with respect to the fully connected version [28,29] is that the finite connectivity allows one to simulate very large sizes, up to $L∼O(10^{4})$, even close to the upper critical dimension, $\rho_{U}=4/3$. Indeed, the model has been used intensively in recent years for the study of the low temperature spin glass phase outside the mean field theory [16,17,19,30].

A very interesting, and still debated, question regards the existence of a spin glass phase transition in the non-mean field region, $\rho_{U}<\rho <2$, when the external magnetic field is present. Standard methods of analysis, used up to now, have provided inconclusive results, often interpreted in opposite ways [19].

We simulated the above model for two values of the long range exponent: ρ=1.2, which lies in the mean field region, and ρ=1.4, which lies in the non-mean field region. The zero-field critical temperatures are $T_{c}\left(h=0\right)=2.34\left(3\right)$ for ρ=1.2 and $T_{c}\left(h=0\right)=1.970\left(2\right)$ for ρ=1.4 [16]. We simulated the equilibrium dynamics of systems of sizes $L\leq 2^{13}$ with field values h=0.1,0.2,0.3 using the parallel tempering method [31,32]. For each value of ρ and *L*, we simulated $O(10^{5})$ different disordered samples.

### 3.2. A New Tool of Analysis Conditioning on the Overlap

Let s and t be the configurations of two real replicas evolved by Glauber dynamics, and suppose *M* independent measurements, taken over many different samples. We define the conditional average of the overlap-overlap correlation function (or 4-point correlation function) as

(16) $$G\left(r|q\right)=\langle q_{0}q_{r}|q\rangle \equiv \frac{\sum_{i,j:|i-j|=r}E\left[s_{i}t_{i}s_{j}t_{j}\;\delta_{qN,s\cdot t}\right]}{\sum_{i,j:|i-j|=r}E\left[\delta_{qN,s\cdot t}\right]}\;,$$

where E[(⋯)] stands for the empirical average over the *M* measurements, δ is the Kronecker delta, and $s\cdot t\equiv \sum_{k=1}^{N}s_{k}t_{k}$ (here the system size N=L because we have a d=1 model). G(r|q) actually depends also on the system size *L* and the temperature *T*, but we avoid making this explicit to keep notation lighter. The standard correlation function is obtained taking the average over the conditioning variate

(17) G(r)=∫dqP(q)G(r|q),

where $P\left(q\right)=E\left[\delta_{qN,s\cdot t}\right]$ is the probability distribution of the overlap.

More precisely the correlation function, G(r), defined above is the total correlation function and can be expressed as a linear combination of the usual connected correlation functions defined in the replicon and longitudinal sectors: $G\left(r\right)=2G_{\mathrm{SG}}\left(r\right)-G_{L}\left(r\right)$ [33]. In order to obtain the two connected correlations separately, we should simulate four replicas instead of two, and the analysis would become much more bothersome. However, this is not really needed since an eventual spin glass phase transition makes both G(r) and $G_{\mathrm{SG}}\left(r\right)$ decay critically.

The rationale beyond this conditional averaging is in the observation [21,24] that measurements of a spin glass model in a field may have very large fluctuations, even in the paramagnetic phase. These large fluctuations are associated with very atypical overlap values: in the samples that one can simulate with presently available computer resources, the distribution of the overlap, P(q), has a tail in the region of small and even negative overlaps; this tail will eventually disappear in the thermodynamic limit, but is responsible for the large finite size corrections. Conditioning on the overlap, we expect G(r|q) to have much weaker fluctuations, so its estimate in the thermodynamic limit should be less problematic. Indeed, we expect that, under the hypothesis of replica equivalence [34,35], the conditional correlation G(r|q) should be self-averaging. The main source of fluctuations would remain in the P(q), which is, however, very much studied in the literature; moreover, in the paramagnetic phase and in the thermodynamic limit $P\left(q\right)=\delta \left(q-q_{\mathrm{EA}}\right)$, and we just need to estimate $q_{\mathrm{EA}}$.

According to what we discussed in Section 2, we expect that, even in the paramagnetic phase, the model may have a spin glass phase transition lowering the value of the conditioning overlap *q*. In order to detect this phase transition, we can look for a critical overlap value, $q_{c}$, such that critical scaling holds in the conditioned susceptibility χ(q) at $q=q_{c}$. To obtain the latter, we Fourier transform the conditioned correlation function

(18) $$\hat{G}\left(k|q\right)=\int dr\;e^{ikr}G\left(r|q\right)\;.$$

By definition $\hat{G}\left(0|q\right)=q^{2}$ and so the best way to extract the large distance behavior of G(r|q) is to look at $\hat{G}\left(k_{\min}|q\right)$, with $k_{\min}=2\pi /L$. Thus, we define the susceptibility as

(19) $$\chi \left(q\right)\equiv \hat{G}\left(k_{\min}|q\right)\;.$$

The model under study has power-law decaying interactions, and this leads to the free propagator [29]

(20) $$\hat{G}{\left(k\right)}^{-1}\propto m^{2}+k^{\rho -1}\;.$$

The η exponent is not renormalized in the non-mean field region ($\rho_{U}<\rho <2$) and takes the value η=3-ρ, so 2-η=ρ-1. Therefore, at criticality ($q=q_{c}$), we expect

(21) $$\chi \left(q_{c}\right)\propto \left\{\begin{array}{cc} L^{\rho -1} & \;\mathrm{for}\;\rho >\rho_{U}=4/3\;(\mathrm{non}-\mathrm{mean}\;\mathrm{field})\;,\\ L^{1/3} & \;\mathrm{for}\;\rho \leq \rho_{U}=4/3\;(\mathrm{mean}\;\mathrm{field})\;. \end{array}\right.$$

With the exponent η known, the value of $q_{c}$ can be obtained from the crossing of the curves $\chi \left(q\right)/L^{2-\eta }$ (or $\chi \left(q\right)/L^{1/3}$ in the mean field regime) plotted as a function of *q*. Notice that, for $q<q_{c}$, we expect a power law divergence of χ(q) as a function of the lattice size, maybe with another power, and for $q>q_{c}$, the susceptibility χ(q) becomes a constant.

The approach of finding the phase transition varying *q* is equivalent, via a Legendre transform, to studying a Hamiltonian with a coupling term, H+ϵqN, and to characterizing the possible phase transitions in the extended three-dimensional parameter space (T,h,ϵ). Notice that, via the Legendre transform, there is a relation at the phase transition between the critical values $\epsilon_{c}\left(T,h\right)$ and $q_{c}\left(T,h\right)$. Our assumption that η=ρ-1 relies on the assumption that the renormalization group transformation has only two fixed points, controlling the phase transitions with h=0 and with h≠0, respectively. Under this hypothesis, the entire critical surface (except the critical point at h=0) belongs to the same universality class.

We recall that, in the one-dimensional long range order model, there is no renormalization of the wave function; thus, the η exponent is known exactly, even in the presence of a magnetic field: η=ρ-1.

Finally, we remark that this phase transition in *q* (or equivalently in ϵ) was characterized in [36] for the three-dimensional Edwards-Anderson model with binary couplings and a zero magnetic field, and below the critical temperature: in that case, $q_{c}=q_{\mathrm{EA}}$ for all $T<T_{c}$, according to the analytical study presented in this paper.

### 3.3. Numerical Results

We present some results for ρ=1.2 (which belong to the mean field region) for comparison and the main results for ρ=1.4, which are in the interesting non-mean field region and far from the lower critical dimension. The lower critical dimension of the model is $\rho_{L}=2$ in the absence of a field [30], but $\rho_{L}$ could decrease in a field [37].

**Figure 6:**
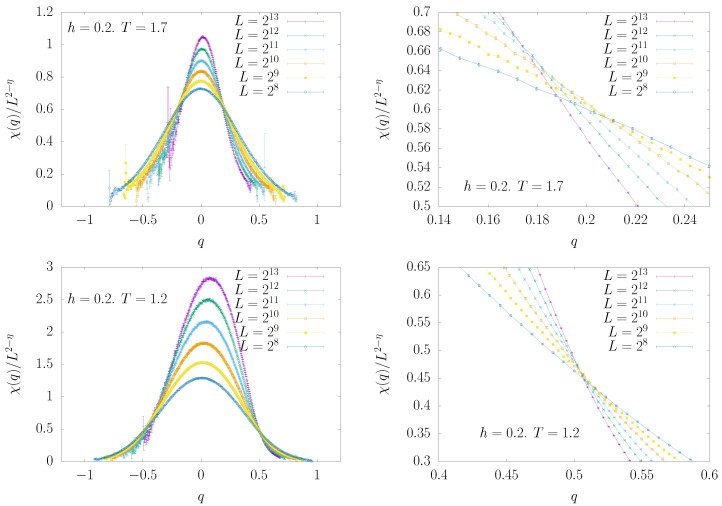
$\chi \left(q\right)/L^{2-\eta }$ versus *q* for ρ=1.4 (non-mean field region) and six different lattice sizes. Data in the upper panels have been measured with T=1.7 and h=0.2 and belong to the paramagnetic phase [16], thus showing that a transition to a spin glass phase can be induced merely by decreasing the overlap between the replicas. In the bottom panels, near or inside the thermodynamic spin glass phase, T=1.2 and h=0.2. The crossing point of the curves for different lattice sizes is always very neat, as can be appreciated from the panels on the right that zoom in on the crossing region.

In Figure 6, we plot the scaled susceptibilities, $\chi \left(q\right)/L^{2-\eta }$, as a function of the conditioning overlap *q*. The two plots are for ρ=1.4, h=0.2, and T=1.7 (top) and T=1.2 (bottom). We remind the reader that $T_{c}\left(h=0\right)≃1.97$ for ρ=1.4 [16]. All the curves, corresponding to sizes from $L=2^{8}$ to $L=2^{13}$, do intersect at well defined values of $q_{c}$, obtained by means of an analysis based on the fit of the data to a cubic spline polynomial taking into account the statistical errors. It is worth noting that the higher temperature, T=1.7, is certainly in the paramagnetic phase, according to the previous estimate of $T_{c}=1.2\left(2\right)$ for h=0.2 [16]. Nonetheless, lowering the overlap to atypical values, smaller than the thermodynamically dominant value $q_{\mathrm{EA}}$, clearly leads to a phase transition at $q_{c}$, in agreement with the solution of the SK model.

We remind the reader that all the measurements have been taken during a standard Monte Carlo simulation, with no condition at all on the overlap among the two replicas. The conditioning on the overlap is imposed only during off-line analysis. The fact that curves in Figure 6 have good statistics in a broad *q* range and become noisy only at the boundaries is a consequence of the very broad support of P(q). Therefore, in some sense, the present analysis is exploiting in a positive way the large fluctuations in the overlap that are present in spin glass models in a field. One may complain that, if the P(q) would become narrow, then the present analysis would fail. But in that case, fluctuations are tiny and the standard analysis (without the conditioning) would provide a reliable answer.

From the crossing point of the scaled susceptibilities shown in Figure 6, we obtain reliable estimates for $q_{c}$. The estimate of $q_{\mathrm{EA}}$ can be obtained in two different ways as long as $T\geq T_{c}\left(h\right)$

- from the peak location in P(q), and
- from the crossing points of the cumulative functions $x\left(q\right)\equiv \int_{-1}^{q}dq^{\prime }\;P\left(q^{\prime }\right)$.

**Figure 7:**
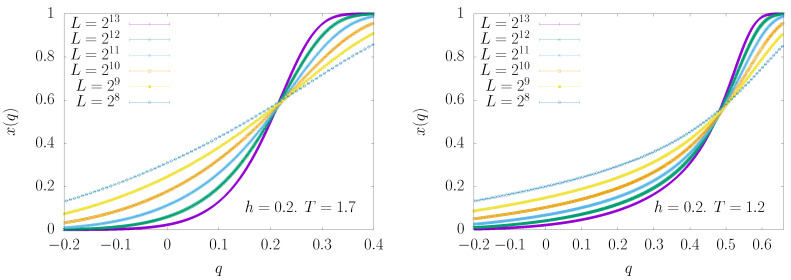
The cumulative probability distribution x(q) versus *q* for ρ=1.4 (non-mean field region), h=0.2, and two values of the temperature: T=1.7 (**left panel**) and T=1.2 (**right panel**). The estimate for $q_{\mathrm{EA}}$ comes from the crossing of these curves.

We found that the second method presents much weaker finite size corrections; thus, we used it to estimate $q_{\mathrm{EA}}$ (see Figure 7). It is worth noting that this second method, although much more accurate as long as $T\geq T_{c}\left(h\right)$, does not return the correct result for $T<T_{c}\left(h\right)$. Indeed, in the latter case, the median value estimated from the crossing of the cumulative functions x(q) is slightly lower than the location of the peak in the P(q). Nonetheless, for the purpose of locating an eventual critical temperature $T_{c}\left(h\right)$, a reliable estimate of $q_{\mathrm{EA}}$ in the region $T\geq T_{c}\left(h\right)$ is enough.

**Figure 8:**
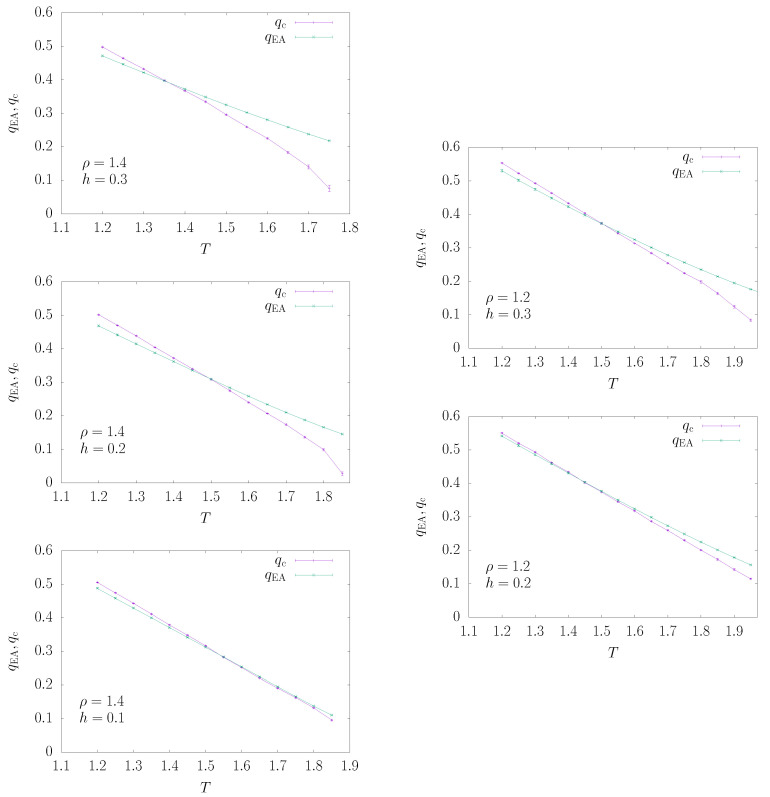
Behavior of $q_{c}\left(T\right)$ and $q_{\mathrm{EA}}\left(T\right)$ for ρ=1.4 (**top-left panel** with h=0.3, **middle-left panel** with h=0.2 and **bottom-left** with h=0.1) in the non.mean field regime and ρ=1.2 (**top-right panel** with h=0.3 and **bottom-right panel** with h=0.2) in the mean field regime. The crossing (or merging) of the curves identifies the thermodynamic phase transition to the spin glass phase (dAT line) because $q_{c}<q_{\mathrm{EA}}$ holds in the paramagnetic phase. Data shown are for the largest sizes ($L=2^{12}$ and $L=2^{13}$).

We show in Figure 8 the estimates of $q_{c}$ and $q_{\mathrm{EA}}$ obtained for ρ=1.4 (left panels) and ρ=1.2 (right panels) with different values of the magnetic field and the largest sizes ($L=2^{12}$ and $L=2^{13}$). As long as $q_{\mathrm{EA}}>q_{c}$ in the thermodynamic limit, the model is not critical and belongs to the paramagnetic phase. Exactly when $q_{\mathrm{EA}}=q_{c}$, the model satisfies critical scaling in the thermodynamic limit; i.e., it belongs to the critical dAT line $T_{c}\left(h\right)$. For $q_{\mathrm{EA}}<q_{c}$, we are below $T_{c}\left(h\right)$; thus, the estimate of $q_{\mathrm{EA}}$ is no longer correct (most probably the correct estimate of $q_{\mathrm{EA}}$ is $q_{c}$ itself).

With the aim of estimating the critical dAT line $T_{c}\left(h\right)$, one should compute the crossing points between the $q_{\mathrm{EA}}\left(T\right)$ and $q_{c}\left(T\right)$ curves shown in Figure 8. For fields large enough (e.g., h≥0.2 for ρ=1.4), the crossing of the $q_{\mathrm{EA}}\left(T\right)$ and $q_{c}\left(T\right)$ curves is very clear; thus, the identification of the critical dAT temperature $T_{c}\left(h\right)$ is highly reliable. On the contrary, for very small fields (e.g., h=0.1 for ρ=1.4), the $q_{\mathrm{EA}}\left(T\right)$ and $q_{c}\left(T\right)$ curves tend to merge, rather than cross each other; thus, the estimate of $T_{c}\left(h\right)$ is noisy. This comes with no surprises given that we expect the two curves to coincide in the h=0 limit. In this case, we adopt the rule of estimating the critical temperature as the highest temperature at which the difference is compatible with zero within one standard deviation (and this may provide a bias towards too large values).

We observe also that the analysis works much better for ρ=1.4 than for ρ=1.2, and this is good news, given that we are mainly interested in locating the dAT line in the non-mean field regime; the data for ρ=1.2 are shown mainly for comparison purposes, but the actual estimates of the critical point in this case are very noisy, as already noted in [16].

In order to highlight how stable the analysis is and to check for finite size corrections, we computed the curves $q_{\mathrm{EA}}\left(T\right)$ and $q_{c}\left(T\right)$ from the data of only two sizes, namely *L* and 2L. In practice, $q_{\mathrm{EA}}$ is obtained from $x_{L}\left(q_{\mathrm{EA}}\right)=x_{2L}\left(q_{\mathrm{EA}}\right)$, while $q_{c}$ satisfies $\chi_{L}\left(q_{c}\right)/L^{2-\eta }=\chi_{2L}\left(q_{c}\right)/{\left(2L\right)}^{2-\eta }$. The *finite size estimate* of the critical temperature corresponds to the crossing of $q_{\mathrm{EA}}\left(T\right)$ and $q_{c}\left(T\right)$, obtained from sizes *L* and 2L. We report these estimates in Table 1. Finite size corrections are smooth and under control, except for the smallest fields (but this is expected given the observation that, at small fields, estimates of $T_{c}\left(h\right)$ are much more noisy).

## 4. Conclusions

In this work, we studied, both analytically and numerically, the phase transition from the paramagnetic to the spin glass phase that takes place in a spin glass model when two real replicas are forced to have an overlap smaller than the equilibrium one.

We have solved the SK model close to its critical point (under the so-called truncated approximation) and have shown analytically that, in the paramagnetic phase in a field (h>0 and $T_{c}\left(h\right)<T<T_{c}\left(0\right)$), lowering the overlap $p_{d}$ between two real replicas leads to a phase transition at $p_{d}^{*}$ where the symmetry between replicas is spontaneously broken. In the whole paramagnetic phase, the inequality $p_{d}^{*}<q_{\mathrm{EA}}$ holds, while $p_{d}^{*}=q_{\mathrm{EA}}$ identifies the critical dAT line.

We have also performed an analysis of data from Monte Carlo simulations of a proxy of a finite-dimensional spin glass model conditioning on the value of the overlap between the two simulated replicas. We have cleanly estimated a value $q_{c}$ of the overlap where the model undergoes a phase transition and it is natural to identify the numerically estimated $q_{c}$ with the analytically predicted $p_{d}^{*}$. The comparison of $q_{c}$ with the typical overlap $q_{\mathrm{EA}}$ turned out to be a very reliable way of estimating the critical dAT line $T_{c}\left(h\right)$.

The technique presented in this paper to characterize the dAT line relies on the fact that the phase transition is continuous. Hence, if other kinds of phase transitions (dynamical, first order, etc.) were present (as suggested by Höller and Read [38]), we could miss them. We consider this new scenario very interesting, and we are considering studying it, but with a different numerical approach.

As far as we know, this phase transition is only present in spin glasses. The same computation presented in this paper on the Curie-Weiss model shows no phase transition on ϵ for temperatures in the paramagnetic phase.

In conclusion, we have provided solid evidence for the existence of a critical dAT line in a finite-dimensional spin glass model in a field by using a new tool of analysis inspired by the presence of a phase transition in the overlap between two real replicas.

## Figures and Tables

**Table 1:** Values of the critical temperature obtained from the crossing points of the curves $q_{\mathrm{EA}}\left(T\right)$ and $q_{c}\left(T\right)$, which have been computed using data from lattices *L* and 2L. The left table is for ρ=1.4 (non-mean field regime), and the right table is for ρ=1.2 (mean field regime). In the row labeled FSSA, we report the critical temperatures obtained in [16] using finite size scaling analysis.

ρ=1.4				ρ=1.2		
$\log_{2}L$	h=0.1	h=0.2	h=0.3	$\log_{2}L$	h=0.2	h=0.3
	$T_{c}$	$T_{c}$	$T_{c}$		$T_{c}$	$T_{c}$
8	1.88(1)	1.56(6)	1.31(4)	8		1.47(10)
9	1.89(3)	1.44(6)	1.39(3)	9	1.36(5)	1.38(5)
10	1.85(1)	1.47(2)	1.40(1)	10	1.4(1)	1.43(4)
11	1.40(3)	1.53(1)	1.39(3)	11	1.48(5)	1.47(3)
12	1.57(9)	1.51(1)	1.37(1)	12	1.51(5)	1.53(2)
FSSA	1.67(7)	1.2(2)		FSSA	1.4(2)	1.5(4)
